# The H.O.U.S.E. classification: a novel endoscopic retrograde cholangiopancreatography (ERCP) complexity grading scale

**DOI:** 10.1186/s12876-017-0583-z

**Published:** 2017-03-09

**Authors:** Greger Olsson, Urban Arnelo, Fredrik Swahn, Björn Törnqvist, Lars Lundell, Lars Enochsson

**Affiliations:** 1 Division of Surgery, CLINTEC, Karolinska Institutet, Stockholm, Sweden; 2 Department of Surgery, Ryhov County Hospital, Jönköping, S-551 85 Sweden; 3 Center for Digestive Diseases, Karolinska University Hospital, Huddinge, Stockholm, S-141 86 Sweden

**Keywords:** ERCP, Complications, Pancreatitis

## Abstract

**Background:**

Endoscopic retrograde cholangiopancreatography (ERCP) is a technically challenging endoscopic procedure, harboring a wide range of complexities within every single investigation. Classifications of the complexity of ERCP have been presented, but do not include modern endoscopic treatment modalities. In order to be able to target resources and compare the results of different endoscopic centers, a new complexity grading system for ERCP is warranted. This study launches a new complexity grading scale for ERCP–the H.O.U.S.E.-classification.

**Methods:**

The medical record of every patient undergoing ERCP 2009–2011 at the Karolinska University Hospital was reviewed, regarding the complexity of the procedure, and categorized into one out of three-grades in the HOUSE classification system, and concomitantly graded according to the Cotton grading system. All ERCP-procedures were also registered in the Swedish registry for gallstone surgery and ERCP (GallRiks) and correlations between the grading systems and procedure related variables as well as outcomes were made.

**Results:**

Between 2009 and 2011, 2185 ERCPs were performed at the Karolinska University Hospital, Huddinge. One thousand nine hundred fifty-four of those were index-ERCPs. Another 23 patients were excluded due to lack of postoperative complication registrations, leaving 1931 ERCP procedures to be analyzed. The procedure times were 40 ± 0.7, 65 ± 1.5 and 106 ± 3.2 min, respectively (HOUSE 1–3). The corresponding pancreatitis rates were 3.4, 7.0 and 6.8% and the postoperative complication rates 11.1, 15.7 and 12.8%, respectively.

**Conclusions:**

The HOUSE-classification is a novel grading scale for ERCP-complexity. The system can be implemented in clinical practice to allocate resources and allow the comparisons of results between different endoscopic centers. Further studies are warranted to further sharpen this instruments validitity and general clinical relevance.

## Background

Endoscopic retrograde cholangiopancreatography (ERCP) is a technically challenging endoscopic procedure, where severe complications cannot be avoided [1–3]. Risk factors behind the development of complications are; the experience of the endoscopist [4–11], case volume of procedures [4, 5, 7–12], the indication for the procedure [7–9] as well as the technical complexity [7–9]. Additional factors affecting outcome are those associated with altered anatomy after gastric surgery, while others are more unpredictable, like a giant duodenal diverticula [13, 14] or a small papilla requiring a precut sphincterotomy for access. Hence some risk factors cannot be anticipated but the majority can be defined in advance and in the ideal world there is room available for prophylactic, preventive measures to be taken to minimize the risk profile for each individual patient. Classifications of the complexity of ERCP have been presented [15, 16], but do not include modern endoscopic treatment modalities. Cotton and coworkers [6] presented a classification system where experienced endoscopists were asked to grade different endoscopic procedures according to their complexity to which was added also their own experience. A median value of their scores was thereafter filed, building up a grading system for the complexity of the ERCPs. Accordingly, this scale represents the only complexity grading scale established during the last decade but it is entirely “eminence-based”. Moreover, available scoring systems in the field of endoscopic complexity grading [6, 15, 16] have never been validated and have also become outdated due to introduction of new diagnostic and treatment facilities. This is of course problematic in itself, but the current situation also hampers the communication of outcomes in clinical practice but obviously so regarding the presentation of research outcomes. There are several additional reasons for introducing a new complexity grading scale in ERCP. Such a grading system has the potential to function as an aid in an ERCP-education program, to find the right level in a step-wise training curricula [15]. It may facilitate the planning and allocation of resources and the amount of time required for a certain procedure. It may also offer a solid background for adequate charging of endoscopic procedures between different healthcare systems and providers. Finally, it may be critically important to assist in defining the right level of competence and experience of the individual endoscopist in patients who are planned to be referred to a tertiary referral-center.

The objectives of the present study were therefore to define, test and validate a new classification system for ERCP with the ambition to address the defined unmet needs of today and in the foreseeable future.

## Methods

### The GallRiks registry

The Swedish Registry of Gallstone Surgery and ERCP (GallRiks) is a nationwide web-based quality registry for gallstone surgery and ERCP [17]. The registry was established in 2005 and has now reached a level, where almost all Swedish hospitals include their cholecystectomies and ERCPs. This registry is approved by the Swedish Surgical Society and is supported by the Swedish Board of Health and Welfare and is based on an internet platform with safe online data registration. All procedures and intra-operative complications are registered on-line by the physicians performing the procedures. The 30-day overall postoperative complication rates as well as ERCP-specific complications like pancreatitis, cholangitis and bleeding due to endoscopic sphincterotomy are registered by dedicated coordinators at each participating hospital.

The database has been validated, indicating a complete (97.3%) concordance between the medical records and the corresponding information captured and filed in the database of ERCP cases [18]. Since the start of the registry, an increasing number of hospitals have joined and attaining now more than 90% coverage and compliance of all ERCPs performed in Sweden [18].

**Fig. 1:**
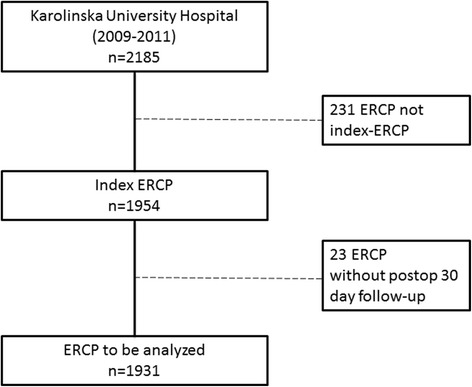
A flow chart depicting the respective ERCP procedures included in the analyses

From the GallRiks registry all ERCPs performed at the Karolinska University Hospital, Huddinge, between 2009 and 2011, were analyzed (Fig. 1). The complication rate for each individual ERCP procedure was monitored from GallRiks and classified accordingly to the HOUSE classification system.

### The HOUSE-classification

Initially the HOUSE-classification (Table 1) was developed and launched into clinical practice to gain financial reimbursement and control of the increasing costs for endoscopic devices used as well as the prolonged procedure times required to complete the expanding complexity of ERCP examinations done at the Karolinska University Hospital Huddinge, which over time has evolved into a national tertiary-referral center for advanced endoscopy. Concomitantly there was a continuous demand for the establishment of a more accurate system for comparisons between different centers, both regarding results as well as post-procedural complication rates. The well merited opinion was that the more complex that the procedures became the greater was the risk that the results were marred by higher complication rates just because of the complexity of the case-mix among patients leading to higher costs. The original database was scored based on the perceived complexity of each procedure and classified into three groups, where group one represented the least complex procedures and group three the most complex (Table 1). One of the aims of the grading was that HOUSE class 1 procedures would represent the least complex routine procedures as represented by those being performed at every hospital performing ERCP in Sweden; extraction of common bile duct stones, relief of obstructive jaundice due to periampullary cancer and intraoperative rendez-vous ERCP. The HOUSE class 2 represents the technically more advanced procedures mainly performed at the county hospitals like ERCP for intrahepatic stones, multiple metal and plastic stenting and ERCP for Primary Sclerosing Cholangitits. The HOUSE class 3 is represented by ERCP procedures demanding extra resources like intraductal cholangioscopy (SpyGlass Direct Visualization System, Boston Scientic Corp, Natick, Mass), double-balloon ERCP for Roux-en-Y operated patients or confocal endoscopy, all procedures being performed at the tertiary referral-centers (Table 1). The database of the HOUSE-classification was then compared with corresponding data from the GallRiks’ database concerning complications in general and pancreatitis rates in particular.

**Table 1:** Description of the different HOUSE-classes

HOUSE class 1
Diagnostic ERCP
Endoscopic sphincterotomy
Single stone (<10 mm)
Plastic stent subhilar
Brush cytology
Multiple stones or stone >10 mm
Metal stent
Plastic stent above hilus
Intraoperative rendez-vous ERCP
HOUSE class 2
Intrahepatic stone
Multiple metal/plasticstents
ERCP specifically pancreatic
Intrahepatic interventions
All patients with PSC or liver Tx
Prophylactic pancreatic stent
“Caged” papilla
ERCP with ESWL
HOUSE class 3
All precut-incl pancreatic sphincterotomy
Spy-Glass
Mother-Baby Scopy
EHL
Lithotripsy (pancreatic)
Multiple pancreatic stent
Papillectomy
Confocal endoscopy
PTC- or EUS-rendez-vous.
B2, Roux-en Y, Whipple, via enteroscopy, GBY-op

The classification is referred to as the HOUSE-classification, which is an abbreviation of the first letter of the name of the hospital (Huddinge) followed by the first letters of the creators’ names (Olsson, Urban, Swahn, and Enochsson).

### Cotton classification

The outcomes of the different ERCP procedures were also classified according to the established classification systems for ERCP procedures; i.e. the Cotton complexity grading of endoscopic procedures [6].

### Statistical analysis

Descriptive data for cannulation success rates, patients suffering complications or not and procedure times were displayed using mean for continuous variables or percentages for categorical variables. Postoperative complications in total and specified (pancreatitis and postoperative bleeding) were calculated within each HOUSE class and compared using Pearson Chi square-test with HOUSE 1 used as reference group. Differences in mean- procedure times between the different HOUSE classes were analyzed using Student’s *t*-test. A p-value <0.05 was regarded as significant. Statistical analyses were carried out using JMP® version 12.1.0 (64-bit) (SAS Institute Inc).

## Results

Two thousand one hundred eighty-five ERCP procedures at Karolinska University Hospital, Huddinge were registered in GallRiks between 2009 and 2011. Of these, 1954 procedures were index-ERCPs, the first ERCP in every treatment episode, and accordingly included in the subsequent analyses. In 23 cases complete 30-day follow-up data were not captured why these were excluded and resulting in 1931 ERCP procedures being analyzed (Fig. 1). The medical records of all remaining patients were reviewed and the intra- as well as post-procedural complications and cannulation success rates were recorded from the GallRiks Registry into every single HOUSE class (HOUSE 1, n = 1124; HOUSE 2, n = 541; HOUSE 3, n = 266). Demographics and indications of the respective HOUSE classes are given in Table 2. The pre-procedural pancreatitis rate in HOUSE class 3 was significantly lower than that of HOUSE class 2 (*P* = 0.0235) but not compared to HOUSE class 1.

**Table 2:** Demographics and indications of the different HOUSE-classes

	HOUSE 1	HOUSE 2	HOUSE 3
Gender			
Female (%)	53.4	41.4	47.7
Male (%)	46.6	58.6	52.3
Age^a^ (year)	63.8 ± 0.5	53.3 ± 0.7	55.8 ± 1.2
ASA 1–2 (%)	64.7	58.0	67.3
	Indications (%)		
Acute pancreatitis	3.0	4.1	1.1
Cholangitis	3.1	4.3	4.9
Chronic pancreatitis	1.4	9.6	10.9
Intraoperative diagnosis	0.5	0.0	0.0
Malignancy	15.0	9.6	9.0
Obstructive jaundice	13.9	9.4	6.0
Other	7.1	16.5	31.6
Scheduled control	11.1	15.7	4.1
Sec. prophylax biliary pancreatitis	1.3	0.6	0.0
Stentdysfunction	2.8	7.0	0.8
Susp. bile leakage	3.8	1.9	1.5
Susp/known CBDS	36.0	7.2	12.8
Susp/known PSC	0.8	14.1	17.3

^a^Mean ± SEM

**Fig. 2:**
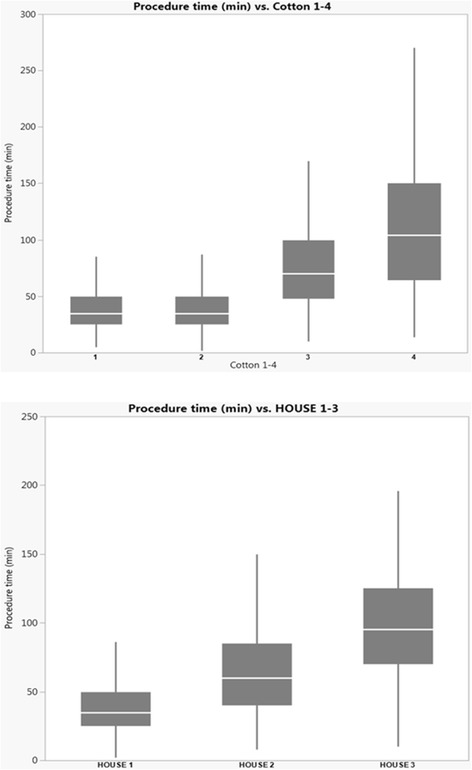
The correlation between the ERCP procedure times (min) and the respective classes of the two different complexity grading systems (HOUSE, Cotton)

**Fig. 3:**
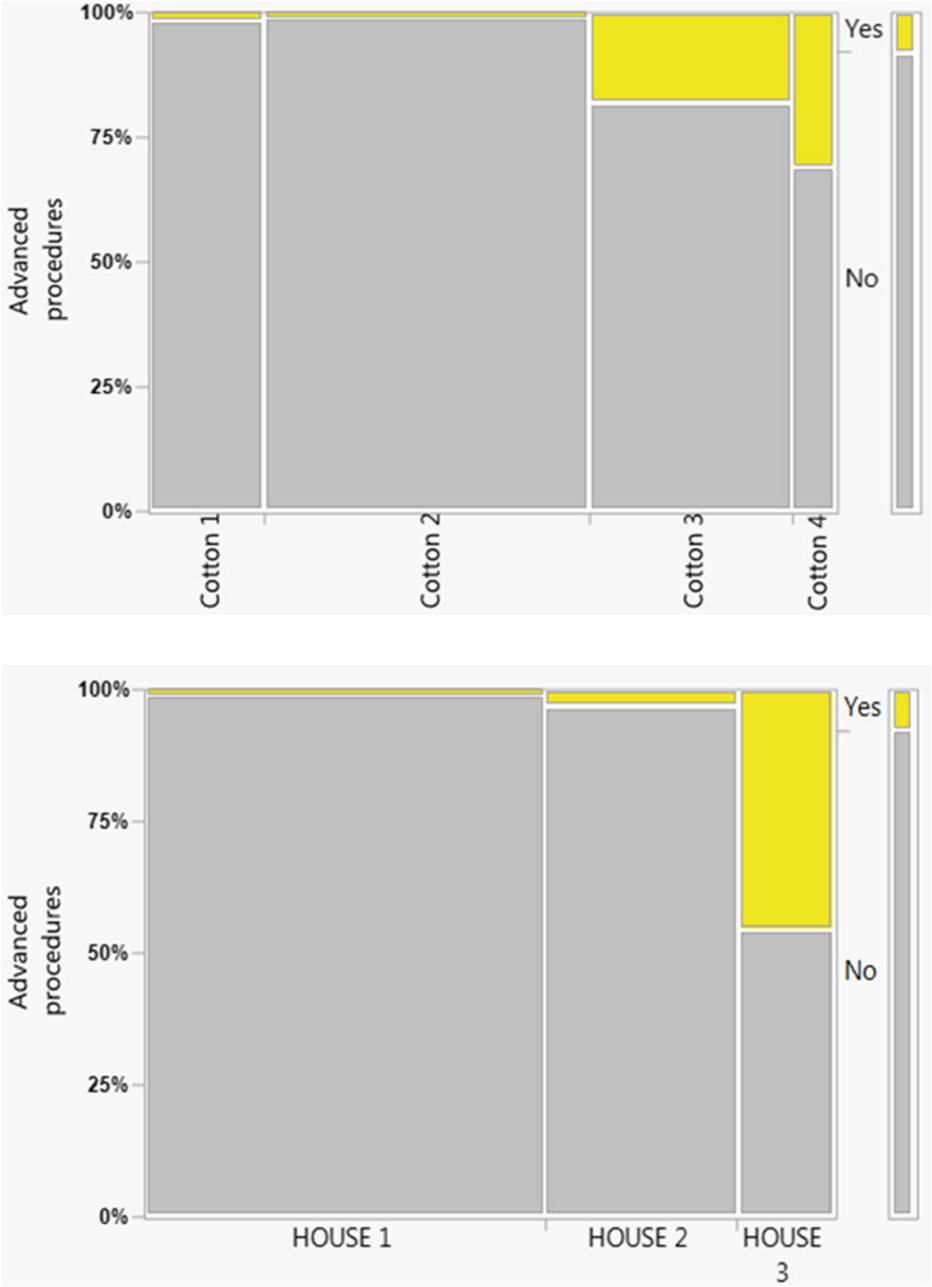
The relation of advanced procedures in the respective classes of the two different complexity grading systems (HOUSE, Cotton)

The mean procedure time in HOUSE 1 was (40 ± 0.7 min) whereas the procedure times increased significantly in HOUSE 2 (65 ± 1.5 min) and HOUSE 3 (106 ± 3.2 min), respectively (Table 3). The correlation between the ERCP procedure times (min) and the respective classes of the two different complexity grading systems (HOUSE, Cotton) are given in Fig. 2. However, bile duct cannulation rates were significantly higher in the HOUSE 2 and 3 groups probably because previous sphincterotomy had been done to a much larger extent in these two groups. More advanced procedures had also, by definition, been done especially in the HOUSE 3 group (Table 3 and Fig. 3).

**Table 3:** Procedure related variables

	TOTAL	HOUSE 1	HOUSE 2	HOUSE 3	*P*
	n = 1931	n = 1124	n = 541	n = 266	
Procedure time (min) (mean ± SEM)	55.9 ± 0.9	40 ± 0.7	65 ± 1.5	106 ± 3.2	<.0001
Deep bile duct cannulation (%)^a^	92.7	90.9	95.8	95.1	0.0009
Previous sphincterotomy (%)	30.4	22.3	42.5	39.5	<.0001
Advanced procedures (%)	7.7	1.0	3.1	45.5	<.0001

^a^136 excluded where the bile duct was not intended to cannulate

**Fig. 4:**
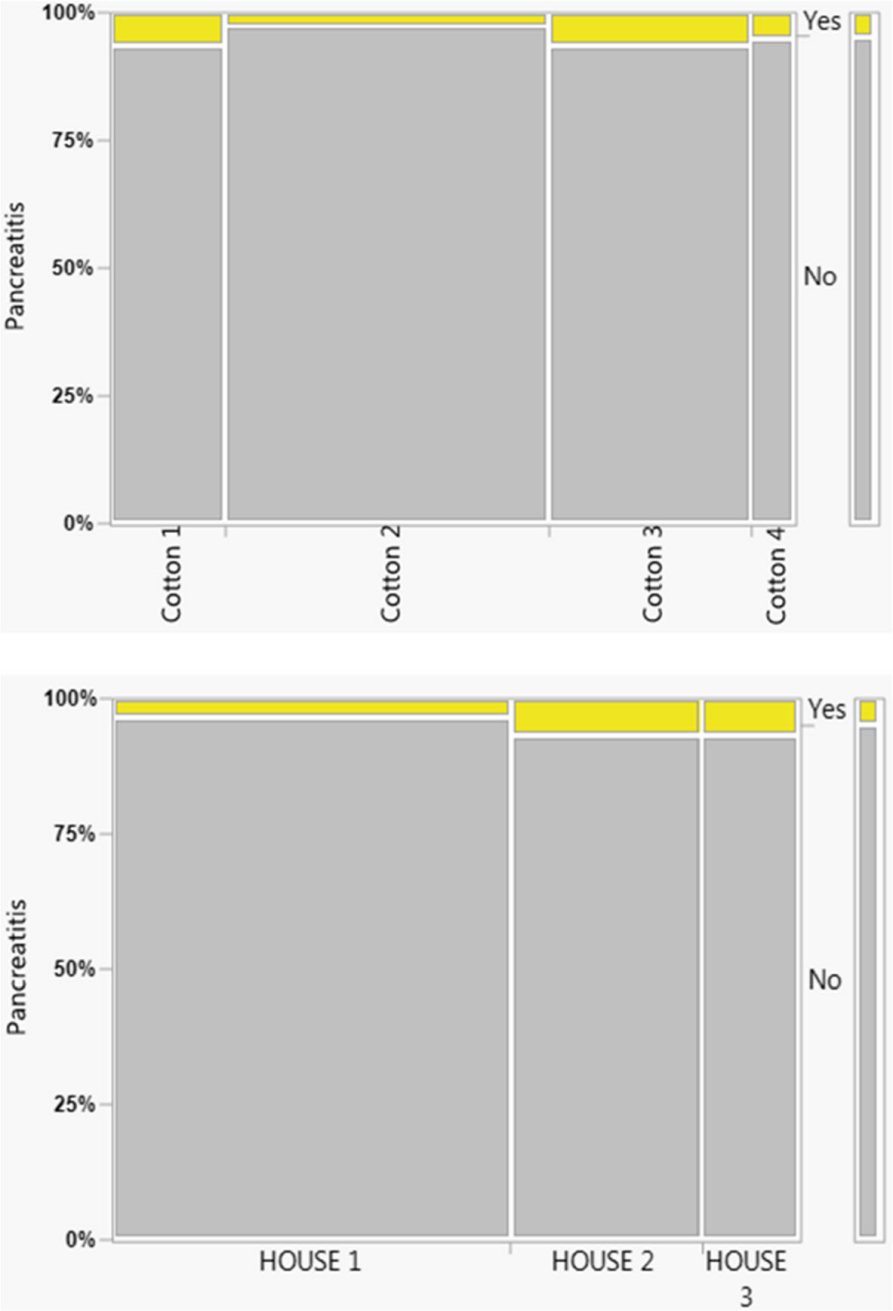
The pancreatitis frequency of the respective classes of the two different complexity grading systems (HOUSE, Cotton)

The overall intraoperative adverse event rate was 4.2% and 244 of the 1931 patients (12.6%) experienced postoperative complications. The overall post ERCP pancreatitis (PEP) rate was 4.9% and the incidence of postoperative bleeding was 2.5% (Table 4). The overall postoperative adverse event rate, at the 30-day follow-up, was 11.1%. in HOUSE class 1. With increasing HOUSE-classifications we recorded a significant increase in the postoperative adverse event rates, as illustrated by 15.7 and 12.8% in HOUSE 2 and 3, respectively (Table 4). The PEP incidence was 3.4% in HOUSE class 1 which increased significantly to 7.0 and 6.8% in HOUSE 2 and 3, respectively (Table 4). The pancreatitis frequency by the respective classes, as scored by the two different complexity grading systems (HOUSE, Cotton), are given in Fig. 4. We were unable to detect any differences between the HOUSE groups regarding sphincterotomy bleeding risk nor in abscess formation rates (Table 4).

**Table 4:** Intra- and postoperative adverse events of the different HOUSE-classes

	TOTAL	HOUSE 1	HOUSE 2	HOUSE 3	*P*
	n = 1931	n = 1124	n = 541	n = 266	
	Intraoperative (%)				
Intraoperative (all)	4.2	4.2	3.9	5.3	0.6489
Bleeding	1.2	1.7	0.4	0.8	0.0519
Extravasation of contrast	1.8	0.9	3.0	3.0	0.0027
	Postoperative (%)				
Postoperative (all)	12.6	11.1	15.7	12.8	0.0305
Pancreatitis	4.9	3.4	7.0	6.8	0.0016
Bleeding	2.5	2.9	1.9	1.9	0.3247
Infection with abscess	0.8	1.1	0.6	0.5	0.3792

## Discussion

The main finding concerning this new HOUSE-classification is that it co-varies well to the complexity of the ERCP procedures as well as to procedure time and, to some extent, to adverse events. The HOUSE-classification is also applicable to and in line with modern endoscopic treatment procedures in ERCP. This indicates that the HOUSE-classification could be used in clinical practice to optimize resource planning for each individual ERCP procedure.

In the complexity-grading scale developed by Schutz [16], no differences in complication rates were seen relating to the complexity grading of the ERCP, except for the success-rates of the procedures. Since radiological and endoscopic techniques have developed after the introduction and establishment of this grading scale, to the continued implementation of these criteria in modern endoscopic treatment is problematic, e.g. a categorization system has to incorporate procedures regardless of whether those are diagnostic or therapeutic. This represents an important limitation since the diagnostic procedures to a large extent have been abandoned, after the introduction of the MRI-technique. The Morriston Hospital Grading Scale by Ragunath et al. [15], was mainly developed for educational purposes. More importantly these investigators were unable to demonstrate significant differences in complication rates between the different complexity grades. The complexity grading system, as launched by Cotton et al. [6], where the classification system was presented based on experienced endoscopists’ personal opinion concerning the complexity of different endoscopic procedures. The calculated median values of these experts’ opinion scores, created the grading system. Accordingly, this was not generated out of a clinical prospective validation process but entirely on eminence-based opinions. Hereby we present a classification system that can be used in clinical daily practice and which correlates to the duration of each individual procedure and post-interventional complications. The calculations and estimates were constructed on a critical review of the clinical courses of close to 2000 ERCP procedures at the Karolinska University Hospital, carried out during a defined timeperiod.

There are several arguments in favor of the introduction of a validated complexity classification system. One of these is to target and allocate adequate resources to where they are needed, e.g. to schedule the right amount of time in the endoscopy suite and/or the appropriate competence and equipment necessary for a specified procedure. Another argument in favor of a corresponding classification can be that it fosters and endorses education and training. Hereby both the trainee and the supervisor could select the right level of procedure complexity, adjusted to the trainee’s current skills and educational program. A third argument is that such a classification system can offer a relevant and precise tool to guide the more complex ERCP-procedures directly to the tertiary referral-centers. Such a grading system may also simplify and enhance the accuracy of the billings of endoscopic procedures harmonizing between different health care systems and providers. A new classification would facilitate the option for intra- and inter-individual comparisons within and between different endoscopic centers regarding case mix, success- and complication rates in a more objective and constructive way. Last but not least clinical research obviously requires well validated instruments to be used when introducing various means through which outcomes can be more safe and effective.

However, there are apparent limitations confined to the study design in the form of selection bias. The study was performed in a tertiary referral center where more complex procedures are over-represented compared with many other endoscopy units. Furthermore, one important driving force behind the development of the classification scoring system was to gain control of the increasing costs for endoscopic procedures. Thus, some of the variables contained within the score are not apparent prior to the actual examination (e.g. if multiple stents were introduced or if there is a “caged” papilla). With the further validation and implementation of the HOUSE classification it might well have to be modified to make it even more clinically relevant and it has also to be tested in other clinical environments, as represented by the non-referral centers [4, 10–12]. Another aspect that has to be taken into consideration is that Karolinska University Hospital is a teaching hospital in endoscopy and it is well known that in an ERCP training situation there is an increased risk for complications [8], diluting the success rates and prolonging the actual procedure times. These aspects warrant the attention of further studies, possible additional modifications and validation of the HOUSE classification in a non-educational endoscopic center.

The HOUSE classification appears to be easy to implement in clinical praxis and facilitates the planning of procedure time and the resources needed for the endoscopy room. It harbors the potential to facilitate education and the endoscopist’s individual training curriculum. However, since the HOUSE-score, in its present form, contains some peri-procedural criteria that cannot be predicted in advance it cannot be used as an objective instrument to direct more complex endoscopic procedures towards tertiary referral-centers.

## Conclusions

In this article a new complexity grading scale for ERCP (HOUSE-classification) is described that can be used in daily clinical practice to optimize resources and allow the comparisons of results between different endoscopic centers. However, further studies are warranted to further sharpen this instruments validitity and general clinical relevance.

## Abbreviations

Deep bile duct cannulation: is defined as deep cannulation of the bile duct with subsequent contrast injection
ERCP: Endoscopic retrograde cholangiopancreatography
H.O.U.S.E.-classification: ERCP complexity grading scale named after the first letter of the name of the hospital (Huddinge) followed by the first letters of the creators’ names (Olsson, Urban, Swahn, and Enochsson)
Index-ERCPs: are defined as the first ERCP-procedure for each respective patient per in-hospital treatment period
Intra- or postoperative bleeding: are defined as bleeding requiring intervention
Intra-procedural adverse events for ERCP: are defined as bleeding, extravasation of contrast, perforation or any other reason for the ERCP being terminated prematurely
Pancreatitis: is defined as abdominal pain in the presence of an elevated amylase at least 3 times above normal at a time point more than 24 h after terminating the procedure
Post-procedural adverse events: are defined as any complication during the 30-day follow-up period that requires some form of medical or surgical intervention
